# Multiband and Broadband Absorption Enhancement of Monolayer Graphene at Optical Frequencies from Multiple Magnetic Dipole Resonances in Metamaterials

**DOI:** 10.1186/s11671-018-2569-3

**Published:** 2018-05-16

**Authors:** Bo Liu, Chaojun Tang, Jing Chen, Ningyan Xie, Huang Tang, Xiaoqin Zhu, Gun-sik Park

**Affiliations:** 1 School of Mathematics and Physics, Jiangsu University of Technology, Changzhou, 213001 China; 2 Center for Optics and Optoelectronics Research, Collaborative Innovation Center for Information Technology in Biological and Medical Physics, College of Science, Zhejiang University of Technology, Hangzhou, 310023 China; 3 College of Electronic and Optical Engineering and College of Microelectronics, Nanjing University of Posts and Telecommunications, Nanjing, 210023 China; 4 Center for THz-driven Biological Systems, Department of Physics and Astronomy, Seoul National University, Seoul, 151-747 South Korea; 5 State Key Laboratory of Millimeter Waves, Southeast University, Nanjing, 210096 China

**Keywords:** Light absorption, Monolayer graphene, Magnetic dipole resonances, Metamaterials, Plasmonics

## Abstract

It is well known that a suspended monolayer graphene has a weak light absorption efficiency of about 2.3% at normal incidence, which is disadvantageous to some applications in optoelectronic devices. In this work, we will numerically study multiband and broadband absorption enhancement of monolayer graphene over the whole visible spectrum, due to multiple magnetic dipole resonances in metamaterials. The unit cell of the metamaterials is composed of a graphene monolayer sandwiched between four Ag nanodisks with different diameters and a SiO_2_ spacer on an Ag substrate. The near-field plasmon hybridizations between individual Ag nanodisks and the Ag substrate form four independent magnetic dipole modes, which result into multiband absorption enhancement of monolayer graphene at optical frequencies. When the resonance wavelengths of the magnetic dipole modes are tuned to approach one another by changing the diameters of the Ag nanodisks, a broadband absorption enhancement can be achieved. The position of the absorption band in monolayer graphene can be also controlled by varying the thickness of the SiO_2_ spacer or the distance between the Ag nanodisks. Our designed graphene light absorber may find some potential applications in optoelectronic devices, such as photodetectors.

## Background

Graphene, a monolayer of carbon atoms tightly arranged in two-dimensional (2D) honeycomb lattice, was first separated from graphite experimentally in 2004 [1]. Since then, graphene has attracted enormous attentions in the scientific community, partly owing to its exceptional electronic and optical properties, including fast carrier velocity, tunable conductivity, and high optical transparency [2]. As one kind of 2D emerging materials, graphene has promising potentials in a wide variety of fields ranging from optoelectronics [3–6] to plasmonics [7–10], to metamaterials [11–15], etc. Due to its unique conical band structure of Dirac fermions, the suspended and undoped graphene exhibits a universal absorption of approximately 2.3% within the visible and near-infrared regions, which is related to the fine structure constant in a monolayer atomic sheet [16, 17]. The optical absorption efficiency is impressive, considering that graphene is only about 0.34 nm thick. However, it is still too low to be useful for optoelectronic devices such as photodetectors and solar cells, which need considerably higher absorption values for efficient operation.

To overcome this problem, various physical mechanisms [18–43] to enhance absorption of graphene in the visible region have been proposed, which include strong photon localization on the defect layer in one-dimensional (1D) photonic crystals [18, 28, 33, 38], total internal reflection [19, 20, 23, 27], surface plasmon resonances [21, 22, 30, 31, 33], evanescent diffraction orders of the arrays of metal nanoparticles [34], and critical coupling to guided mode resonances [25, 26, 32, 34, 35, 37, 39–41]. Besides the absorption enhancement in graphene, achieving multiband and broadband light absorption in graphene is also important for some graphene-based optoelectronic devices from a practical point of view. But, it is still a challenge, as pointed out in the very recent reports [44–46]. At present, different approaches have been proposed to broaden the bandwidth of graphene absorption in wide frequency range from THz [44–62] and infrared [63–65] to optical frequencies [19, 23, 29, 31, 34–36, 38–40, 43]. Especially, a multi-resonator approach was proven to be a very effective method to resolve the bandwidth limitation of graphene absorption in the THz and infrared regions [45, 46, 62, 63]. In the multi-resonator approach, deep-subwavelength multiple resonators with different sizes are closely packed, which could extend the absorption bandwidth when their resonance frequencies overlap with each other. However, to the best of our knowledge, up to now there are only a few reports on such a multi-resonator approach to obtain multiband and broadband light absorption of graphene in the visible region.

In this work, by employing similar multi-resonator approach, we will numerically demonstrate multiband and broadband absorption enhancement of monolayer graphene in the whole visible wavelength range, which arise from a set of magnetic dipole resonances in metamaterials. The unit cell of metamaterials consists of a graphene monolayer sandwiched between four Ag nanodisks with different diameters and a SiO_2_ spacer on an Ag substrate. The near-field plasmon hybridizations between individual Ag nanodisks and the Ag substrate form four independent magnetic dipole modes, which result into four-band absorption enhancement of monolayer graphene. When the magnetic dipole modes are tuned to be overlapped spectrally by changing the diameters of Ag nanodisks, a broadband absorption enhancement is achieved. The position of the absorption band in monolayer graphene can be also controlled by varying the thickness of the SiO_2_ spacer or the distance between the Ag nanodisks.

## Methods/Experimental

**Fig. 1:**
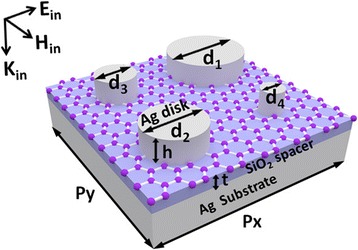
Schematic of metamaterials for multiband and broadband absorption enhancement of graphene at optical frequencies, which are composed of a graphene monolayer sandwiched between four Ag nanodisks and a SiO_2_ spacer on an Ag substrate. Geometrical parameters *p*_*x*_ and *p*_*y*_ are the array periods along the *x* and *y* directions, respectively; *t* is the thickness of the SiO_2_ spacer; *d*_*1*_, *d*_*2*_, *d*_*3*_, and *d*_*4*_ are the diameters of four Ag nanodisks (*d*_*1*_ > *d*_*2*_ > *d*_*3*_ > *d*_*4*_); *h* is the height of the Ag nanodisks. ***E***_in_, ***H***_in_, and ***K***_in_ are the electric field, magnetic field, and wave vector of the incident light, which are along the *x*, *y*, and *z* axes, respectively

The designed metamaterials for multiband and broadband absorption enhancement of graphene at optical frequencies are schematically shown in Fig. 1. The unit cell of the metamaterials consists of a graphene monolayer sandwiched between four Ag nanodisks with different diameters and a SiO_2_ spacer on an Ag substrate. We calculate the reflection and absorption spectra, and the distributions of electromagnetic fields by the commercial software package “EastFDTD, version 5.0,” which is based on finite difference time domain (FDTD) method (www.eastfdtd.com). In our numerical calculations, the refractive index of SiO_2_ is 1.45, and the frequency-dependent relative permittivity of Ag is taken from experimental data [66]. Under the random-phase approximation, the complex surface conductivity *σ* of graphene is the sum of the intraband term *σ*_intra_ and the interband term *σ*_inter_ [67, 68], which are expressed as follows:

1 $$ {\sigma}_{\operatorname{int}\mathrm{ra}}=\frac{ie^2{k}_BT}{\pi {\mathrm{\hslash}}^2\left(\omega +i/\tau \right)}\left(\frac{E_f}{k_BT}+2 In\left({e}^{-\kern0.5em \frac{E_f}{k_BT}}+1\right)\right),{\sigma}_{\operatorname{int}\mathrm{er}}=\frac{ie^2}{4\pi \mathrm{\hslash}} In\left(\frac{2E{}_f-\left(\omega +i/\tau \right)\mathrm{\hslash}}{2E{}_f+\left(\omega +i/\tau \right)\mathrm{\hslash}}\right), $$

where *ω* is the frequency of incident light, *e* is electron charge, *ħ* is reduced Planck constant, *E*_*f*_ is Fermi energy (or chemical potential), *τ* is the relaxation time of electron-phonon, *k*_*B*_ is Boltzmann constant, *T* is temperature in K, and *i* is the imaginary unit. Graphene has an anisotropic relative permittivity tensor of *ε*_*g*_ expressed as

2 $$ {\varepsilon}_g=\left(\begin{array}{ccc}1+ i\sigma /\left({\omega \varepsilon}_0{t}_g\right)& 0& 0\\ {}0& 1+ i\sigma /\left({\omega \varepsilon}_0{t}_g\right)& 0\\ {}0& 0& 1\end{array}\right), $$

where *ε*_*0*_ is the permittivity of the vacuum, and *t*_*g*_ is the thickness of graphene sheet.

## Results and Discussion

**Fig. 2:**
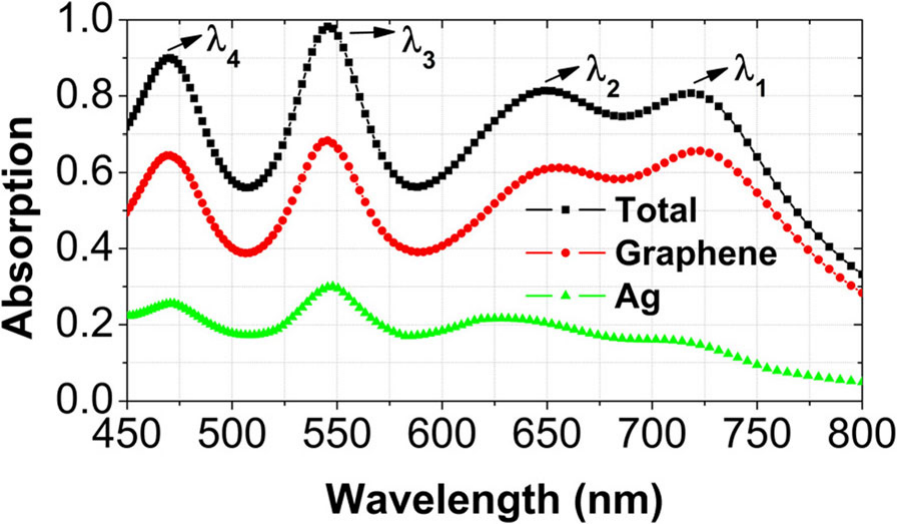
Normal-incidence absorption spectra of monolayer graphene (red circle), Ag (green triangle), and total metamaterials (black square) in the wavelength range from 450 to 800 nm. Geometrical and physical parameters: *p*_*x*_ = *p*_*y*_ = 400 nm, *d*_*1*_ = 140 nm, *d*_*2*_ = 110 nm, *d*_*3*_ = 80 nm, *d*_*4*_ = 50 nm, *h* = 50 nm, *t* = 30 nm, *E*_*f*_ = 0.50 eV, *τ* = 0.50 ps, *T* = 300 K, *t*_*g*_ = 0.35 nm

Figure 2 shows the calculated absorption spectra of graphene, Ag, and total metamaterials at normal incidence. One can clearly see four absorption peaks, whose resonance wavelengths are *λ*_1_ = 722.9 nm, *λ*_2_ = 655.7 nm, *λ*_3_ = 545.5 nm, and *λ*_4_ = 468.8 nm. At four absorption peaks, the light absorption in graphene can reach as high as 65.7, 61.2, 68.4, and 64.5%, respectively. Compared with a suspended monolayer graphene whose absorption efficiency is only 2.3% at optical frequencies [16, 17], the monolayer graphene in our designed metamaterials has an absorption enhancement of more than 26 times. It is also clearly seen in Fig. 2 that the absorbed light is mainly dissipated in graphene rather than in Ag. Moreover, the total absorption at the third peak exceeds 98.5%, very similar to much reported metamaterial electromagnetic wave perfect absorbers [69–75], which have many potential applications such as solar cells [76–81].

**Fig. 3:**
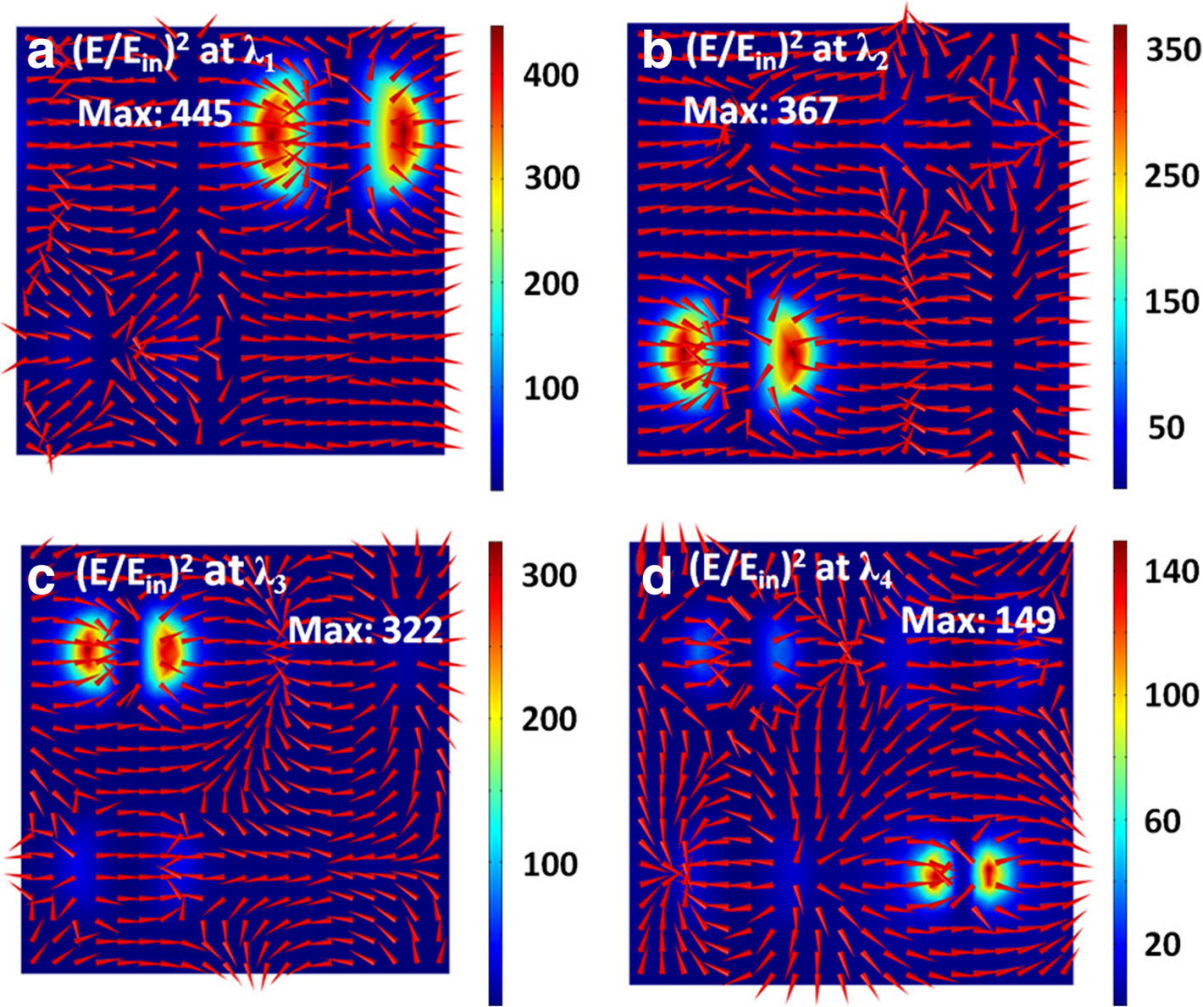
(**a**)-(**d**) Corresponding normalized electric field intensity (*E*/*E*_*in*_) on the xoz plane across the center of the SiO spacer for the resonance wavelengths of λ , λ , λ , and λ labeled in Fig. 2. Red arrows represent the field direction, and colors show the field strength

**Fig. 4:**
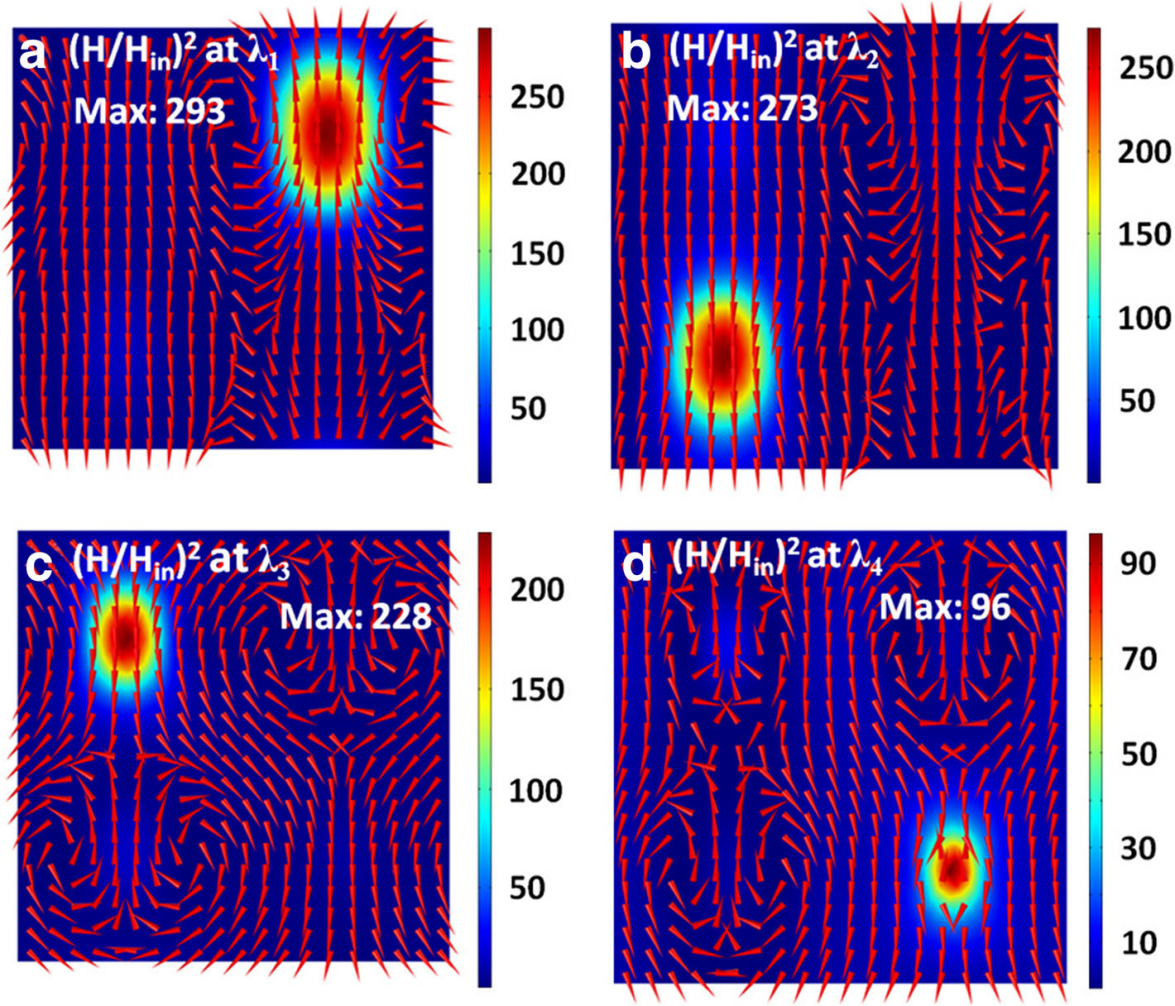
The same as in Fig. 3, but for normalized magnetic field intensity (*H*/*H*_*in*_)^2^

To find the physical origins of above four absorption peaks, Figs. 3 and 4 plot the distributions of electric and magnetic fields at the resonance wavelengths of *λ*_1_, *λ*_2_, *λ*_3_, and *λ*_4_. At the resonance wavelength of *λ*_1_, the electric fields are mainly concentrated near the left and right edges of the first Ag nanodisk with a diameter of *d*_*1*_ (see Fig. 3a), and the magnetic fields are highly confined within the SiO_2_ region under the first Ag nanodisk (see Fig. 4a). Such field distributions correspond to the excitation of a magnetic dipole mode [82–86], which steps from the near-field plasmon hybridization between the first Ag nanodisk and the Ag substrate. At the resonance wavelengths of *λ*_2_, *λ*_3_, and *λ*_4_, the electromagnetic fields have the same distribution properties, but are localized in the vicinity of the second, third, and fourth Ag nanodisks with diameters of *d*_*2*_, *d*_*3*_, and *d*_*4*_, respectively. In short, the excitations of four independent magnetic dipole modes lead to the appearance of four absorption peaks in Fig. 2.

**Fig. 5:**
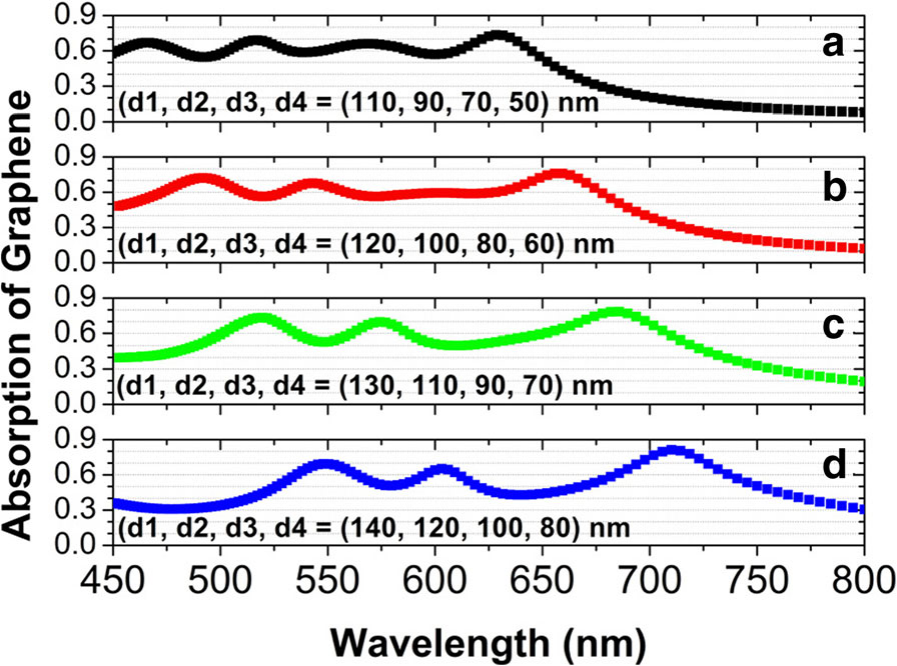
(**a**)-(**d**) Corresponding normal-incidence absorption spectra of monolayer graphene in the wavelength range from 450 to 800 nm with the diameters of four Ag nanodisks are varied, but the other parameters are the same as those in Fig. 2

In our designed metamaterials, the near-field plasmon hybridizations between individual Ag nanodisks and the Ag substrate form four independent magnetic dipole modes, which result into multiband absorption enhancement of monolayer graphene in the visible wavelength range from 450 to 800 nm, with an average absorption efficiency exceeding 50% (please see Fig. 2). The resonance wavelength of each magnetic dipole mode can be conveniently tuned by changing the diameter of the corresponding Ag nanodisk. If the diameters of the Ag nanodisks are varied for the absorption peaks in Fig. 2 to approach one another, a broad high-absorption band of monolayer graphene will be formed. To demonstrate this, Fig. 5a presents the normal-incidence absorption spectra of monolayer graphene, when the diameters *d*_*1*_, *d*_*2*_, *d*_*3*_, and *d*_*4*_ of four Ag nanodisks are equal to 110, 90, 70, and 50 nm, respectively. In this case, a broadband absorption enhancement in the wavelength range from 450 to 650 nm is achieved by the spectral design on the overlapped absorption peaks, with the lowest (highest) absorption efficiency more than 50% (73%). For the diameters of the Ag nanodisks to be increased gradually, this broad high-absorption band is red-shifted, as shown in Fig. 5b, c.

**Fig. 6:**
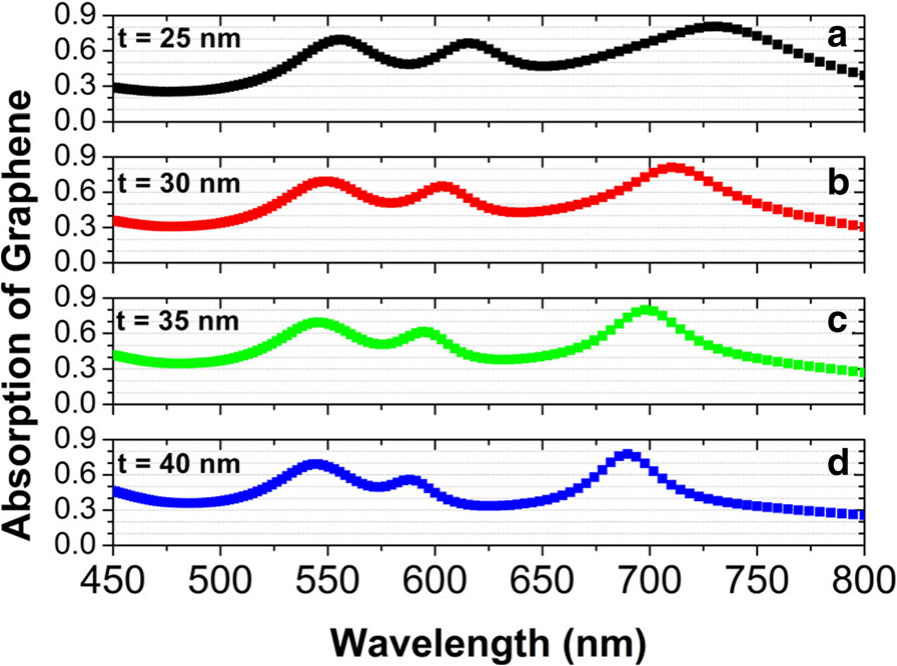
(**a**)-(**d**) Corresponding normal-incidence absorption spectra of monolayer graphene with the thickness of the SiO_2_ spacer increased from 25 to 40 nm in steps of 5 nm. The diameters of the Ag nanodisks are d_1_ = 140 nm, d_2_ = 120 nm, d_3_ = 100 nm, d_4_ = 80 nm, and the other parameters are the same as those in Fig. 2

Besides the diameters of the Ag nanodisks, we can tune the position of the absorption band in monolayer graphene by changing the thickness *t* of the SiO_2_ spacer. Figure 6 shows the normal-incidence absorption spectra in monolayer graphene, for *t* to be increased from 25 to 45 nm. With the increasing *t*, the absorption band in monolayer graphene will have an obvious blue-shift, because the near-field plasmon hybridizations between individual Ag nanodisks and the Ag substrate become weaker and thus magnetic dipole modes are blue-shifted [83].

**Fig. 7:**
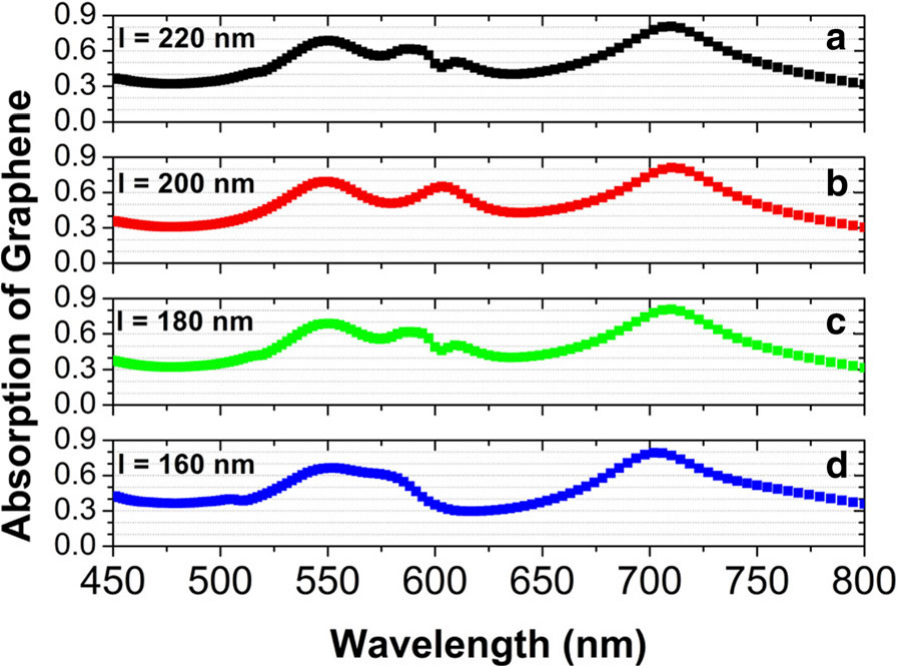
The same as in Fig. 6, but for the center distance l between the nearest-neighbor Ag nanodisks to be decreased from 220 to 160 nm

In the above calculations, the coordinate points of four Ag nanodisks are (±*p*_*x*_ /4, ±*p*_*y*_ /4), so the center distance *l* between the nearest-neighbor Ag nanodisks is 200 nm. By varying *l*, we can also tune the position of the absorption band in monolayer graphene. Figure 7 gives the normal-incidence absorption spectra in monolayer graphene, for *l* to be decreased from 220 to 160 nm. With the decreasing *l*, the absorption band in monolayer graphene is slightly blue-shifted, owing to the plasmon interactions among the Ag nanodisks.

## Conclusions

In this work, we have numerically investigated multiband and broadband absorption enhancement of monolayer graphene at optical frequencies from multiple magnetic dipole resonances in metamaterials. The unit cell of the metamaterials consists of a graphene monolayer sandwiched between four Ag nanodisks with different diameters and a SiO_2_ spacer on an Ag substrate. The near-field plasmon hybridizations between individual Ag nanodisks and the Ag substrate form four independent magnetic dipole modes, which result into multiband absorption enhancement of monolayer graphene in the visible wavelength range. When the magnetic dipole modes are tuned to be overlapped spectrally by changing the diameters of Ag nanodisks, a broadband absorption enhancement is achieved. The position of the absorption band in monolayer graphene can be also controlled, by varying the thickness of the SiO_2_ spacer or the distance between the Ag nanodisks. The numerical results may have some potential applications in optoelectronic devices, such as photodetectors.

## Abbreviations

1D: One-dimensional
2D: Two-dimensional
FDTD: Finite difference time domain
